# Hints on Vulcanite Work

**Published:** 1862-10

**Authors:** Geo. E. Snow

**Affiliations:** Hornellsville, N. Y.


### HINTS ON VULCANITE WORK.

BY DR. GEO. B. SNOW, HORNELLSVILLE, N. Y.

THE following method of substituting new rubber for the old plate (in case of a miss fit), without disturbing the arrangement of the teeth, will be found useful.

Make a moderately thick plaster model in the old plate. Trim it, without removing the plate from it, and varnish its edges. Then oil the fronts of the teeth, plate and model, and cover them with plaster, making a parting between the central incisors. We now have an accurate mold of the labial and buccal surfaces of the teeth and plate. The plaster should extend to the model, which should be notched in such a manner that these "molds" can be accurately readjusted at any time.

After these molds are removed, heat the model and plate sufficiently to soften the rubber, when the teeth can be removed from the plate, and the plate from the model, with ease. Now clear the rubber from between the joints of the teeth. Put a wax plate on the model, adjust the molds, and put the teeth in their places. After waxing them securely to the plate, remove it to a new model, taken from an accurate impression. The joints can now be closed up, the wax trimmed to shape, and the case treated in the usual manner.

This plan will be found preferable, in many cases, to the common one of fastening the rubber plate on a correct model, and investing in the flask at once. The joints can be got together in better shape, and the unsightly appearance of rubber in them avoided in a great measure.

Where the pins are properly bent, or where headed pins are used, it is not always easy to remove the old rubber from the flask. This is especially so in case of deep lower sets. By the method above described, these troubles are avoided, and the little extra time required will be well spent.

There are some remarks in the Register for last April concerning the articulation of rubber plates, in which a difficulty is mentioned, arising from the parts of the flask not being brought together. The difficulty may be avoided, with the common way of getting up molds, by adopting the following precautions :

Ascertain the exact quantity of rubber necessary for the case, (See Register for April last, p. 184,) using but little surplus. Cut a large groove, entirely around the model, if possible, with leaders from the mold at short intervals. Use some kind of a screw purchase to bring the flask together. The Whitney flask is the best in use, so far as this point is concerned.

The rubber should not be packed against the gums of the teeth, where these are ground thin and set close to the model. Pack closely and abundantly around the rivets, and put the rest of the rubber on the palate and in the rim over the gums, and it will find its way behind the gums without fail. Where the alveolar border projects very much, the parting should be opposite the most prominent part of the ridge.

In heating the flasks, preparatory to packing, a small sheet iron stove, standing on the work bench, and heated with gas or alcohol, will be found convenient. It should have a door near the top, to give access to a movable sheet iron muffle, in which the flask can be placed. The muffle should have room at the sides for the hot air to pass around it. The top of the stove should also be movable, so that by removing it and the muffle, a tin dish of suitable form can be inserted, in which water can be heated. This saves all bother of running to cook-stoves, which will not always be found in operation when wanted.

